# Molecular identification of transmembrane protein 68 as an endoplasmic reticulum-anchored and brain-specific protein

**DOI:** 10.1371/journal.pone.0176980

**Published:** 2017-05-04

**Authors:** Ping’an Chang, Christoph Heier, Wenzhen Qin, Liping Han, Feifei Huang, Quan Sun

**Affiliations:** 1 Key Laboratory of Molecular Biology, School of Bio-information, Chongqing University of Posts and Telecommunications, Chongqing, P. R. China; 2 Institute of Molecular Biosciences, University of Graz, Heinrichstrasse 31/II, Graz, Austria; USDA-ARS, UNITED STATES

## Abstract

Acyltransferases catalyze essential reactions in the buildup and remodeling of glycerophospholipids and contribute to the maintenance and diversity of cellular membranes. Transmembrane protein 68 (TMEM68) is an evolutionarily conserved protein of unknown function, that forms a distinct subgroup within the glycerophospholipid acyltransferase family. In the current study we expressed murine TMEM68 for the first time in mammalian cells to characterize its subcellular localization, topology, and possible biological function(s). We show that TMEM68 is an integral membrane protein and orients both, the N- and C-terminus towards the cytosol. Live cell imaging demonstrated that TMEM68 is localized mainly at the endoplasmic reticulum (ER), but not at cellular lipid droplets (LDs). The positioning of TMEM68 at the ER was dependent on its first transmembrane domain (TMD), which by itself was sufficient to target cytosolic green fluorescence protein (GFP) to the ER. In contrast, a second TMD was dispensable for ER localization of TMEM68. Finally, we found that among multiple murine tissues the expression level of TMEM68 transcripts was highest in brain. We conclude that TMEM68 is an integral ER membrane protein and a putative acyltransferase involved in brain glycerolipid metabolism.

## Introduction

Glycerophospholipids are essential structural components of biological membranes, lipoproteins and pulmonary surfactant, and serve as precursors of bioactive signaling lipids, such as platelet-activating factor (PAF) and eicosanoids [1, 2]. The biosynthesis and remodeling of glycerophospholipids relies on a class of enzymes termed acyltransferases, which catalyze the transfer of acyl moieties from acyl-CoA to the glycerol-3-phosphate backbone of glycerophospholipids [3]. *De novo* biosynthesis of glycerophospholipids is initiated by glycerol-3-phosphate acyltransferase (GPAT), which catalyzes the synthesis of lysophosphatidic acid (LPA) from glycerol 3-phosphate by transfer of an acyl moiety from acyl-CoAs. A second acyl moiety is transferred to LPA by 1-acylglycerol-3-phosphate acyltransferase (AGPAT) resulting in the formation of phosphatidic acid (PA). PA is a central intermediate in the biosynthesis of phospholipids, diacylglycerol (DAG), and triacylglycerol (TAG) and can be further metabolized via different enzymatic pathways [4, 5]. Dephosphorylation of PA by PA phosphatase yields DAG as a precursor for the synthesis of TAG, as well as for phosphatidylcholine (PC) and phosphatidylethanolamine (PE) via a metabolic route commonly referred to as the Kennedy pathway. A second metabolic route converts PA to cytidine-diphospho-DAG, the precursor of other glycerophospholipids, such as phosphatidylinositol (PI), phosphatidylglycerol (PG), cardiolipin (CL), and phosphatidylserine (PS) [4, 5].

Glycerophospholipids synthesized by the *de novo* pathway are often further modified through the so-called Lands’ cycle that involves the selective hydrolysis of a fatty acid at the *sn*-1 or *sn*-2 position by a phospholipase A followed by the re-incorporation of acyl moieties by an lysophospholipid acyltransferase (LPLAT) [6,7]. The Lands’ cycle leads to an asymmetric distribution of acyl groups and creates a high diversity of phospholipid species with different structural and functional properties [3, 6]. Typically, phospholipid remodeling via the Lands’ cycle leads to an enrichment of polyunsaturated acyl groups at the *sn*-2 position whereas acyl groups at the *sn*-1 position are usually saturated and monounsaturated. LPLATs have been identified in unrelated protein families including the AGPAT and the membrane bound O-acyltransferase (MBOAT) families [8].

Sequences alignment of several acyltransferases involved in the *de novo* synthesis of phospholipids such as GPAT1, AGPAT1 and AGPAT2, shows that they share a conserved acyltransferase domain (pfam01553), which is characterized by 4 conserved motifs and constitutes the mammalian glycerophospholipid acyltransferase family [9, 10]. Motifs I and IV are involved in catalysis whereas motifs II and III are implicated in substrate binding [11]. The invariant histidine and aspartate in Motif I (HxxxxD) and the proline in Motif IV (xxxxPxx) are important for catalysis [11, 12]. Amino acids important for binding substrates are the phenylalanine and arginine in Motif II (GxxFxxR), and glutamate and glycine in Motif III (xxEGxx) [11, 12]. Based on sequence similarities the glycerophospholipid acyltransferase family can be further divided into seven subfamilies [10]. The first subfamily contains the mitochondrial GPATs and a dihydroxyacetone-phosphate acyltransferase (DHAPAT). The polyglycerophospholipid acyltransferases, LPGAT1 and LCLAT1 as well as AGPAT3-5 belong to the second subfamily. Tafazzin, a transacylase involved in cardiolipin remodeling constitutes subfamily three. Microsomal GPAT3 and GPAT4 form subfamily 4. The LPLATs LPCAT1, LPCAT2, LPCAT2B and LPCAT4 constitute subfamily 5. AGPAT 1 and AGPAT 2 form subfamily 6. Finally, subfamily 7 is distantly related to other subgroups and contains a single protein of unknown function, which is termed transmembrane protein 68 (TMEM68) [10]. In contrast to the glycerophospholipid acyltransferase family, LPLATs of the MBOAT family contain the motifs WD, WHGxxxGYxxxF, YxxxxF and YxxxYFxxH, which are essential for LPLAT activities [13].

Although significant progress has been made in the functional characterization of glycerophospholipid acyltransferases, TMEM68 remains largely uncharacterized and little is known about the subcellular localization, tissue expression, and molecular function of this protein. In order to gain first insights into basic molecular features of TMEM68, we expressed it in mammalian cells and characterized its subcellular localization, topology, and tissue expression pattern. We found that TMEM68 is a polytopic transmembrane (TM) protein at the endoplasmic reticulum (ER) and identified domains critical for membrane association and ER targeting of the protein. Moreover, we demonstrate that among multiple tissues TMEM68 transcript levels are highest in the adult brain. We conclude that TMEM68 is an integral ER membrane protein and a putative acyltransferase involved in brain glycerolipid metabolism.

## Materials and methods

### Materials

African green monkey kidney fibroblast-like COS-7 cells were purchased from the Cell Center of Chinese Academy of Medical Sciences (Beijing, China). The plasmids pEGFP-N3 and pDsRed2-ER were purchased from Clontech (Palo Alto, CA, USA). Q5^®^ site-directed mutagenesis kit was purchased from New England Biolabs (Ipswich, MA, USA). pcDNA™4/HisMax C, TRIzol^®^ reagent, SuperScript^TM^ III First-Strand Synthesis System, Lipofectamine 2000 and HCS LipidTOX™ Deep Red were purchased from Thermo Fisher Scientific (Waltham, MA, USA). pFLAG-CMV-5.1, cell culture reagents, phenylmethylsulfonyl fluoride (PMSF) and proteinase K (PK) were purchased from Sigma-Aldrich (St. Louis, MO, USA). SYBR^®^ RT-PCR Kit and Ex Taq^TM^ HS DNA polymerase, *Bam*H I, *Xho* I, *Eco*R I, and pMD19-T were purchased from Takara (Dalian, China). Mouse monoclonal antibodies anti-GFP (sc-101525, anti-His (sc-8036), anti-FLAG (sc-166384), anti- PDI (sc-376370) and anti-GAPDH (sc-293335), and goat anti-mouse IgG HRP were from Santa Cruz Biotechnology (Santa Cruz, CA, USA). Enhanced chemiluminescence (ECL) reagents were obtained from Pierce Biotechnology (Rockford, IL, USA).

### Animal housing and tissue collection

This study was carried out in strict accordance with the recommendations in the Guide for the Care and Use of Laboratory Animals of Chongqing Science& Technology Commission. The protocol was approved by the Committee on the Ethics of Animal Experiments of Chongqing University of Posts and Telecommunications (Permit Number: 2014–0012). All surgery was performed under ether anesthesia, and all efforts were made to minimize suffering.

Adult male C57BL/6 mice between ages of 12 and 16 weeks were purchased from the Experimental Animal Center of Chongqing Academy of Chinese Materia Medica (Chongqing, China). Adult males were maintained on a regular light-dark cycle and fed a standard laboratory chow diet containing 4.5% wt/wt fat. Mice were sacrificed by ether anesthesia and tissue samples were collected immediately.

### Molecular cloning and mutagenesis

Total RNA from murine brain was isolated using TRIzol^®^ reagent according to the manufacturer’s instructions and then reverse transcribed to cDNA using Oligo (dT)_20_ primers and the SuperScript^TM^ III First-strand Synthesis System for RT-PCR. According to a predicted murine *Tmem68* gene sequence (GenBank accession no. NM_028097), specific forward and reverse primers, 5'-ATGATAGATAACAACCAAACCT-3' and 5'-CTAATGAGCCTTCTGCTCTTTAT-3' were designed to amplify the coding sequence of the *Tmem68* gene. A 50μl PCR reaction volume contained 5μl 10×PCR buffer, 3μl 25 mM MgSO_4_, 4μl 2.5 mM dNTP mixture, 2μl synthesized cDNA template, 1μl 20 μM forward and reverse primers, 0.5μl Ex Taq^TM^ HS DNA polymerase, and 33.5μl sterilized H_2_O. The amplification program was: 94°C for 4 minutes to pre-denature cDNA template; then 35 amplification cycles of 95°C for 15 seconds, 56°C for 30 sec, 72°C for 1 minute to amplify; last 72°C for 10 minutes to extend. PCR products were cloned into pMD19-T- and sequenced. The full-length TMEM68 cds was amplified by PCR using gene-specific primers (Table 1) and then inserted into pcDNA™ 4/HisMax C and pFLAG-CMV-5.1 to generate TMEM68 tagged with His_6_ and FLAG at the N- and C-terminus, respectively. Constructs expressing full-length TMEM68, the first TMD (Residues 51–75, TMD1), the second TMD (Residues 121–145, TMD2) and two TMDs (51–145 residues, TMD1+2) fused with GFP at the C-terminus were generated by PCR amplification of the respective DNA fragments using primer pairs shown in Table 1 and insertion into pEGFP-N3 via the *Eco*R I/*Bam*H I restriction sites. The TMEM68-GFP construct was used as a template for the generation of internal deletion mutants lacking TMD1, TMD2 or TMD1+2. The mutations were generated with primer pairs listed in Table 1 and the Q5^®^ site-directed mutagenesis kit following the manufacturer’s instructions. All plasmids were sequenced to confirm the presence of the desired sequences.

**Table 1:** Primers used to generate TMEM68 constructs.

Construct Name (Coding Sequence)	Primer Name	Primer Sequence (5'-3')
TMEM68-GFP (1–329	TP68F1	GCGAATTCGCCATGATAGATAACAACCAAAC
	TP68R1	CAGGATCCATGAGCCTTCTGCTCTTTAT
TMD1-GFP (51–75)	TM1F	GCGAATTCGCCATGCTGATACTTTTAATACTTCC
	TM1R	CAGGATCCCTTGTAGATATGGAGGAAAA
TMD2-GFP (121–145)	TM2F	GCGAATTCGCCATGGGAGCTGCACTTATAATTTT
	TM2R	CAGGATCCGATGAAAATTTTAGCCATGA
TMD1+2-GFP(51–145)	TM1F	GCGAATTCGCCATGCTGATACTTTTAATACTTCC
	TM2R	CAGGATCCGATGAAAATTTTAGCCATGA
TMEM68-FLAG (1–329)	TP68F1	GCGAATTCGCCATGATAGATAACAACCAAAC
	TP68R1	CAGGATCCATGAGCCTTCTGCTCTTTAT
His_6_-TMEM68 (1–329)	TP68F2	CAGGATCCATGATAGATAACAACCAAAC
	TP68R2	TTCTCGAGATGAGCCTTCTGCTCTTTA
ΔTMD1-GFP (Δ51–75)	ΔTM1F	AGGAAGAATGTATTAAAAGAAGCC
	ΔTM1R	TGGGGTGAAAACCCACAAAAG
ΔTMD2-GFP(Δ121–145	ΔTM2F	CAGAAAGGCAGAACTTGC
	ΔTM2R	TTCTGGTATCTTTTCCATCC
ΔTMD1+2-GFP(Δ51–145)	ΔTM2F	CAGAAAGGCAGAACTTGC
	ΔTM1R	TGGGGTGAAAACCCACAAAAG

### Computational sequence analysis of TMEM68

The amino acid sequence of TMEM68 was deduced from the obtained cDNA sequence using DNAman. The protein family database (PFAM), an online database containing collections of protein domains and families (http://pfam.xfam.org/), was used to analyze protein domains [14]. The conserved domains within TMEM68 were searched by using CD-search online (http://www.ncbi.nlm.nih.gov/Structure/cdd/wrpsb.cgi) [15]. TMHMM (http://www.cbs.dtu.dk/services/TMHMM-2.0/) were used to predict transmembrane domains (TMD). Protein subcellular localization was predicted by WoLF PSORT program (http://wolfpsort.org/). Sequence alignments were generated using Clustal Ω and phylogenetic clustering was performed using the unweighted pair group method with arithmetic mean. The following NCBI accession numbers were used for sequence retrieval and alignments: NP_001032642.1, hAGPAT3; NP_060831.2, hAGPAT5; NP_872357.2, hLYCAT1; NP_064518.1 1, hAGPAT4; NP_060309.2, hLPCAT2; NP_079106.3, hLPCAT1; NP_001273586.1, hTMEM68; NP_001243351.1, hGPAT3; NP_001231878.1, hGPAT1; NP_997211.2, hGPAT2; NP_705841.2, hLPCAT4; NP_001012745.1, hAGPAT2; NP_848934.1, hGPAT4; NP_116130.2, hAGPAT1; NP_055051.1, hDHAPAT; NP_000107.1, hTAZ; NP_001307737.1, hLPGAT1; NP_477513.2, hMGAT1; NP_001013597.1, hAWAT1; NP_001002254.1, hAWAT2; NP_940914.1, hDGAT2-L6; NP_079374.2, hMGAT2; NP_115953.2, hDGAT2; NP_835470.1, hMGAT3; NP_001171439.1, mLYCAT1; NP_001128301.1, mLPGAT1; NP_443747.2 1, mAGPAT3; NP_080920.2 1, mAGPAT4; NP_081068.1 1, mAGPAT5; NP_663351.3, mLPCAT1; NP_766602.1, mLPCAT2; NP_081875.1, mLPCAT2B; NP_001167018.1, mTAZ; NP_001074558.2, mGPAT2; NP_032175.2, mGPAT1; NP_034452.3, mDHAPAT; NP_997089.1, mLPCAT4; NP_082373.1, mTMEM68; NP_766303.1, mGPAT3; NP_080488.1, mAGPAT2; NP_001156851.1, mAGPAT1; NP_061213.2, mGPAT4; NP_080989.2, mMGAT1; NP_001107556.1, mDGAT2-L6; NP_001074605.1, mAWAT1; NP_808414.2, mAWAT2; NP_803231.1, mMGAT2; NP_080660.1, mDGAT2; NP_648888.1, dmCG4729; NP_730158.1, dmCG4753; NP_001036265.1, dmCG32699; NP_477432.3, dmCG8766; NP_572828.1, dmCG3812; NP_609231.2, dmCG17608; NP_651597.1, dmCG5508; NP_525010.1, dmCG4625; NP_608409.1, dmCG15450; NP_726415.1, dmCG3209; NP_610317.1, dmCG1941; NP_001096954.1, dmCG34348; NP_610319.2, dmCG1946; NP_610318.1, dmCG1942.

### Expression analysis of TMEM68 in adult tissues

Total RNA was isolated from tissues of 3 male C57BL/6J mice using theTRIzol^®^ reagent and then reverse transcribed to cDNA with random primers as described above. SYBR^®^ real-time quantitative PCR (qPCR) was performed using the MX3000P Real-Time PCR System with *ribosomal protein*, *large*, *P0 (Rplp0*, *36B4)* as a reference gene. Two specific primers, 5′-TCTTGAGGAGTGGTCACTTGT-3′ and 5′-GCAAAGCCTTTACGATTACCCC-3′, were designed for the analysis of TMEM68 expression. The forward and reverse primers for *36B4* were 5′-AGATTCGGGATATGCTGTTGGC3′ and 5′-TCGGGTCCTAGACCAGTGTTC-3′ respectively. Relative expression levels were determined by the ΔΔCt method. Three biological replicates were conducted for each tissue and each biological replicate was technically repeated three times.

### Cell culture and transfection

COS-7 cells were maintained in Dulbecco’s modified Eagle’s medium (DMEM) supplemented with 10% fetal bovine serum and antibiotics in a 37°C incubator with 5% CO_2_ and 95% humidity. The cells were maintained in the logarithmic phase of growth and sub-cultured at 3–4 days intervals. Plasmid DNA was transfected into COS-7 cells using Lipofectamine 2000.

### Membrane preparation

48-h post transfection, COS-7 cells were washed 2 times with ice-cold PBS, collected using a cell scraper, and harvested by brief centrifugation. Cells were homogenized on ice with 15 passages through a 25-gauge hypodermic needle. Nuclei and cell debris were removed by centrifugation at 1,000 *g* for 5 minutes at 4°C. The post-nuclear supernatant was further centrifuged at 100,000 *g* for 45 minutes at 4°C in an Optima^TM^ TLX ultracentrifuge by using a TLA120 rotor (Beckman). Resulting membrane pellets were resuspended in PBS. The protein concentration of the membrane fractions was determined using the BCA Kit (Pierce) according to the manufacturer’s instructions.

### Membrane protein extraction and protease protection assays

Samples of total membranes were adjusted to 1 μg/μl of protein and treated with PBS, 1% SDS, 1% Triton X-100, or 0.1 M sodium carbonate (pH 11.5) in PBS on a rotator at room temperature for 20 min. Samples were centrifuged at 100,000 *g* for 45 min at 4°C and pellets were resuspended in the original volume of PBS. The samples were then mixed with 5×SDS loading buffer and boiled for 5 min. All the samples were subjected to SDS-PAGE and immunoblot analysis.

Aliquots of the membrane fractions (50 μg of protein) dissolved in PBS were treated with 1.0 mg/ml proteinase K (PK) in the presence or absence of 1% Triton X-100 for 30 min at room temperature. The reactions were stopped by adding PMSF at a final concentration of 5 mM. The samples were then mixed with 5×SDS loading buffer and boiled for 5 min. All the samples were subjected to SDS-PAGE and immunoblot analysis.

### Immunoblot analysis

The samples were subjected to SDS-PAGE, transferred to PVDF filters, and subjected to immunoblot analysis as previously described [16]. Antibodies were used at the following dilutions: mouse anti-GFP, 1:4000; anti-His, 1:4000; anti-Flag, 1:4000; anti-PDI, 1:2000; anti-calnexin, 1:2000; anti-GAPDH, 1:5000; anti-mouse IgG, 1:5000.

### Confocal fluorescence microscopy

To assess co-localization with the ER, COS-7 cells were co-transfected with plasmids encoding for TMEM68-GFP or mutants and DsRed-ER. DsRed-ER encodes for DsRed fused N-terminally with the ER targeting sequence of calreticulin and C-terminally with an ER retention sequence (KDEL). 48 hours post transfection the cells were subjected to live cell imaging. To assess co-localization with lipid droplets (LDs), cells were incubated for 16 h in a regular growth medium supplemented with 400 μM oleic acid (OA) complexed to fat-free BSA to increase LDs formation and then fixed for 20 min with 4% para-formaldehyde. LDs were stained by incubating cells with HCS LipidTOX Deep Red (1:500) for 30 min. Fluorescent images were acquired by confocal scanning microscopy with a Leica SP5 laser scanning confocal microscope. All the presented experiments were repeated independently at least 3 times.

### Statistical analysis

Data are generally expressed as mean values ± standard deviation (SD). Groups of data were compared by one-way ANOVA and by post hoc analysis using Student-Keuls method. Error bars indicate SD from three replicates and differences were considered significant at *P*<0.05.

## Results

### Sequence analysis and phylogenetic relationship of murine TMEM68

**Fig 1:**
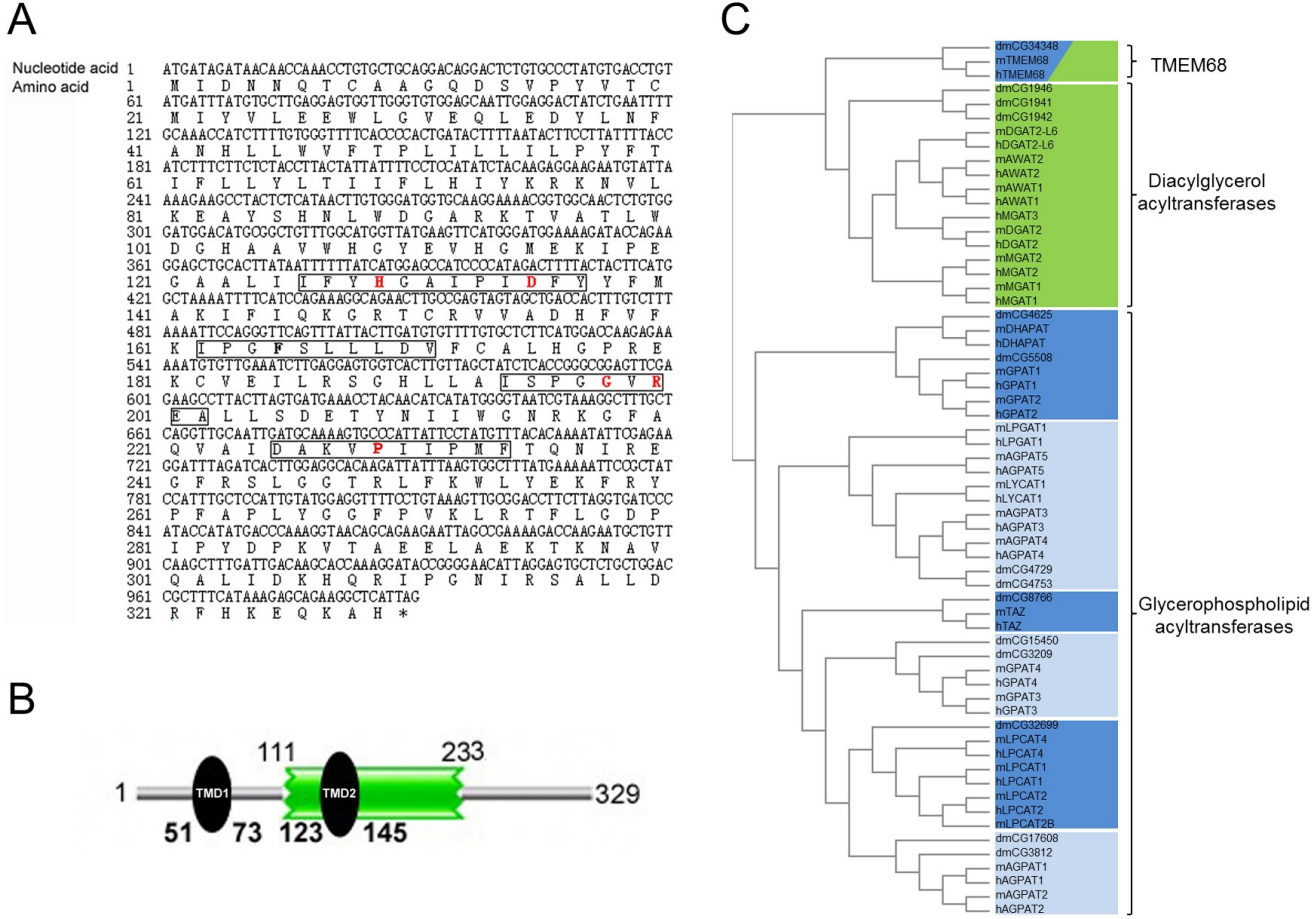
Sequence and protein domains and phylogenetic relationship of murine TMEM68. **(A) The coding sequence and deduced amino acid sequence of the *TMEM68* gene.** The nucleotide and predicted amino acid sequences were countered on the left side. Asterisks represent a stop codon. The putative motif I (residues 126–137) and IV (residues 225–234) are shown in the box containing the conserved active sites residues H129 and D135 as well as P229 respectively, which are typed in red color. Residues in the putative motifs II and III (residues 162–171, and residues 194–202) predicted to be important for substrate binding are typed in bold black and red color. **(B) The protein domain structure of murine TMEM68**. TMEM68 contains a putative acyltransferase domain (residues 111–233) and two transmembrane (TM) domains (residues 51–73, and residues 123–145) shown as green rectangle and black ellipse, respectively. (C) Phylogenetic alignment of murine TMEM68 and other acyltransferases. Protein sequences of glycerophospholipid and diacylglycerol acyltransferase family members (pfam01553 and pfam03982) from human (h), mouse (m) and fruit fly (dm) were aligned using Clustal Ω and clustered using the unweighted pair group method with arithmetic mean.

The predicted coding sequence of murine *TMEM68* was amplified from murine brain cDNA. A polypeptide with 329 amino acids was deduced from the obtained coding sequence (Fig 1A). Sequence analysis using the TMHMM algorithm and the Pfam database of protein domains predicted two putative TMDs (Residues 51–73, and Residues 123–145) and the acyltransferase domains pfam01553 (glycerophospholipid acyltransferase family, residues 111–233) as well as pfam03982 (DAG acyltransferase family, residues 188–253) in the sequence of TMEM68 (Fig 1B). A BLAST search revealed the presence of TMEM68 orthologues in the genome of human (encoded by the *TMEM68* gene) and fruit fly (encoded by the *CG34348* gene), which exhibited 91% and 42% identity to murine TMEM68, respectively. Low similarities between murine TMEM68 and other murine acyltransferases such as 2-acylglycerol O-acyltransferase 1 (MGAT1, 20% identity) and MGAT2 (18% identity, data not shown) were also observed. Further analyses revealed that there are 4 conserved motifs characteristic of the acyltransferase family in the sequence of TMEM68. Residues 129–135 within the putative acyltransferase domain correspond to Motif I with the invariant active sites residues H129 and D135, which could constitute a catalytic dyad (Fig 1A). Although Motifs II and III (residues 162–171, and residues 194–202) in TMEM68 were not well conserved for lacking the typical residues arrangement, they could be putative acyl-acceptor binding pockets. F165 in motif II, G198 and R200 in motif III may be important for substrate binding (Fig 1A). In addition, the conserved proline in the putative Motif IV (residues 225–234)involved in catalysis, exists in murine TMEM68 (Fig 1A). Phylogenetic alignments with glycerophospholipid and DAG acyltransferase family members of human, mouse and fruit fly revealed that murine TMEM68 and orthologous proteins form an evolutionarily conserved subgroup, which is distinct from other acyltransferases (Fig 1C). Taken together, sequence analysis suggests that TMEM68 may be a novel glycero(phospho)lipid acyltransferase localized at cellular membranes.

### TMEM68 is an integral membrane protein

**Fig 2:**
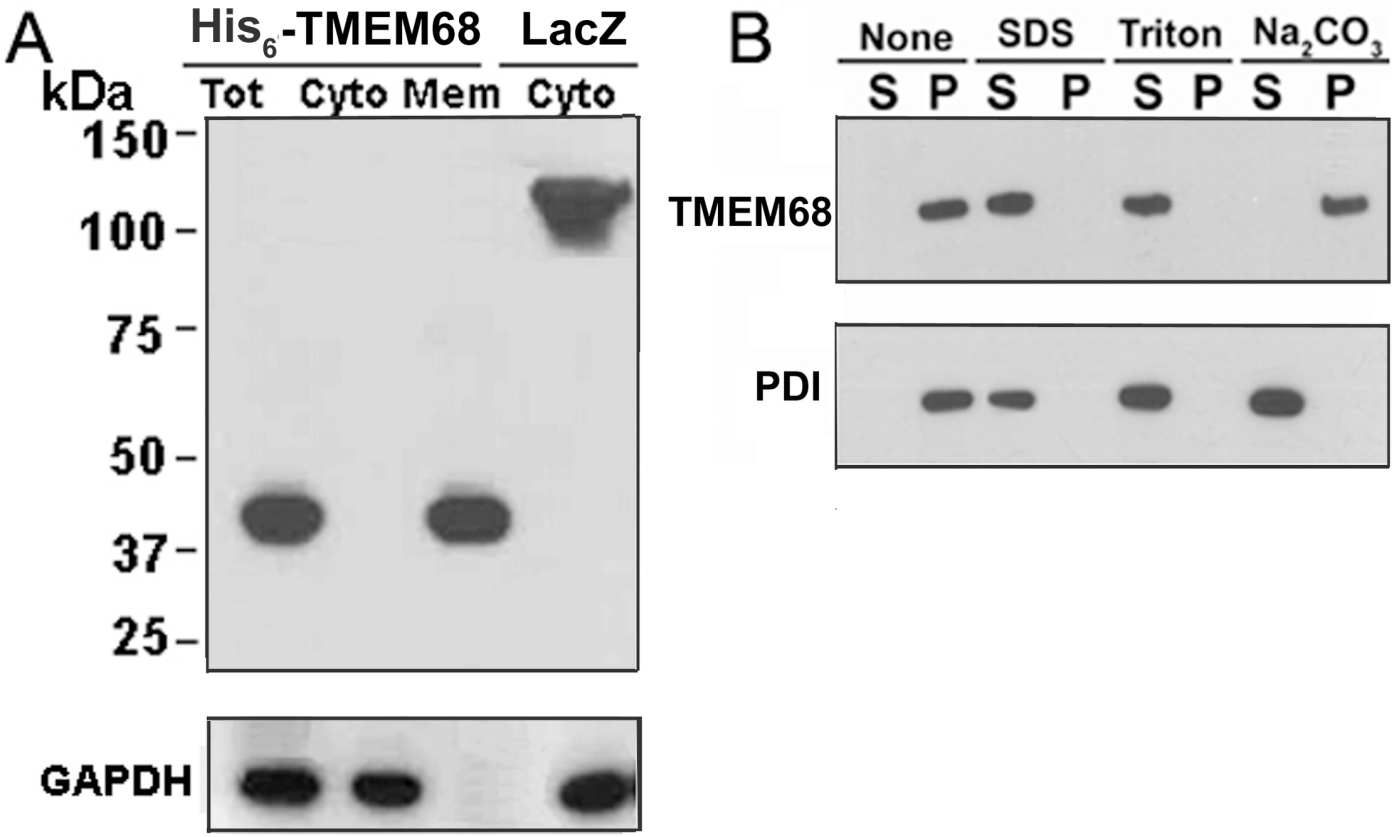
Membrane association of TMEM68. (A) Cellular post-nuclear supernatants (PNS) obtained from COS-7 cells expressing His_6_-TMEM68 were fractionated by centrifugation (100,000 *g*) into membrane (mem, pellet) and cytosolic (cyto, supernatant) fractions. Protein detection was performed by immunoblotting using antibodies against the N-terminal His_6_-tag and GAPDH. (B) Membrane fractions were prepared from COS-7 cells expressing His_6_-TMEM68 and treated with PBS, 1% SDS, 1%Triton X-100, or 0.1 M sodium carbonate (pH 11.5). After incubation, samples were separated into pellet (P) and supernatant (S) fractions and the presence of His_6_-TMEM68 and PDI were detected by immunoblotting using anti-His_6_ and anti-PDI antibodies.

Biochemical analysis was performed to certify whether TMEM68 is associated with cellular membranes. To this end, post-nuclear supernatants of COS-7 cells expressing His_6_-tagged TMEM68 (His_6_-TMEM68) were separated into cytosolic (100,000 *g* supernatant) and membrane fractions (100,000 *g* pellet) and immunoblotting analysis was performed. As shown in Fig 2A, in the whole cell extract His_6_-TMEM68 was detected at about 42 kDa in accordance with the deduced molecular weight sum of TMEM68 (deduced MW 38 kDa) and the tag (deduced MW 3.6 kDa). Moreover, His_6_-TMEM68 was detected exclusively in the membrane fraction, whereas His_6_-β-galactosidase, a transiently expressed control protein, was exclusively recovered in the cytosolic fraction.

Next, we addressed the mode of membrane association of TMEM68. Membrane fractions obtained from COS-7 cells expressing His_6_-TMEM68 were incubated with various solubilizing agents, and separated into soluble (supernatant, S) and insoluble (pellet, P) fractions by ultracentrifugation. As shown in Fig 2B, TMEM68 was found exclusively in the pellet fraction when samples were incubated with aqueous buffer alone. TMEM68 was released into the soluble fraction in the presence of 1% SDS or 1% Triton X-100. In contrast, addition of 0.1 M sodium carbonate, which is known to release peripheral and luminal membrane proteins, failed to solubilize TMEM68 (Fig 2B). These results demonstrate that TMEM68 is an integral membrane protein. As an ER luminal membrane protein, PDI is a control of vesicle integrity. When exposed to PBS alone, PDI was found in the pellet fraction while PDI was solubilized from the pellet into the supernatant fraction by the incubation with SDS, Triton X-100, or sodium carbonate (Fig 2B).

### N- and C- Terminus of TMEM68 are oriented towards the cytosol

**Fig 3:**
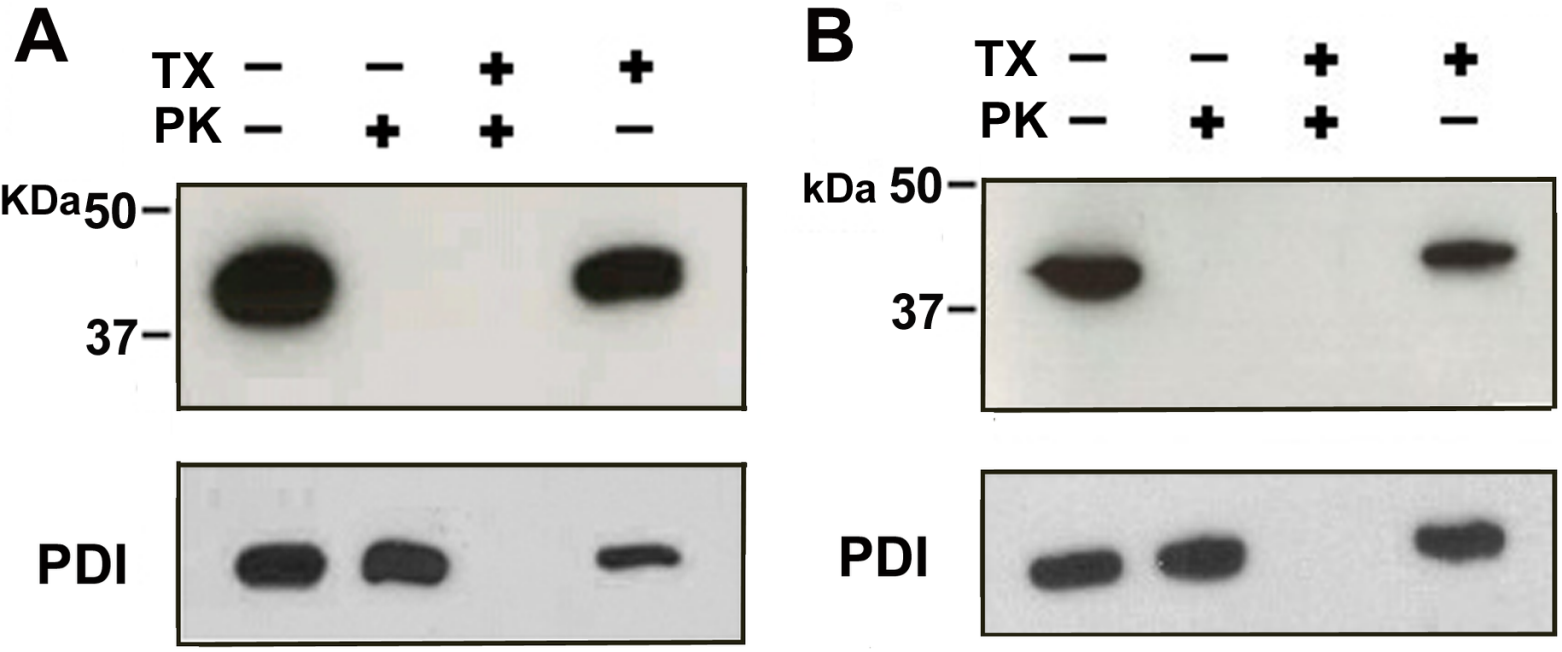
Membrane orientation of the N- and C-terminus of TMEM68. Total membranes obtained from the COS-7 cells expressing His_6_-TMEM68 (A) and TMEM68-FLAG (B) were incubated in the absence or presence of proteinase K (PK) and/or 1% Triton X-100 (TX) and subjected to immunoblotting using antibodies against His_6_, FLAG, and PDI.

In order to assess the membrane topology of TMEM68 protease protection assays were performed. Intact membrane vesicles isolated from COS-7 cells expressing TMEM68 with an N-terminal His_6_-tag (His_6_-TMEM68) or a C-terminal FLAG tag (TMEM68-FLAG) were treated with proteinase K in the absence or presence of Triton X-100 and then analyzed by immunoblotting. In the absence of detergent or proteinase K, both His_6_-TMEM68 and TMEM68-FLAG were recovered exclusively in the pellet fractions (Fig 3). TMEM68-FLAG migrated at about 39 kDa in accordance with the deduced MW sum of TMEM68 and FLAG tag. Treatment of membrane vesicles with proteinase K rendered both the His_6_ and FLAG epitopes undetectable by immunoblotting analysis indicating proteolysis of both the N- and C-terminus. Proteolysis of TMEM68 by proteinase K was independent of the presence of detergent whereas incubation of membrane vesicles with detergent in the absence of proteinase K did not affect detection of the epitopes (Fig 3). To control the integrity of membrane vesicles, the luminal ER protein PDI was detected by immunoblotting. As shown in Fig 3, PDI was clearly protected against proteolysis from proteinase K unless the membrane vesicles were disrupted by the treatment with Triton X-100 indicating that membrane vesicles were indeed sealed. Taken together these results indicate that both, the N- and C-terminal ends of TMEM68 are exposed to the cytosol and suggest that TMEM68 has an even number of TMDs.

### TMEM68 is localized at the ER, but not at LDs

**Fig 4:**
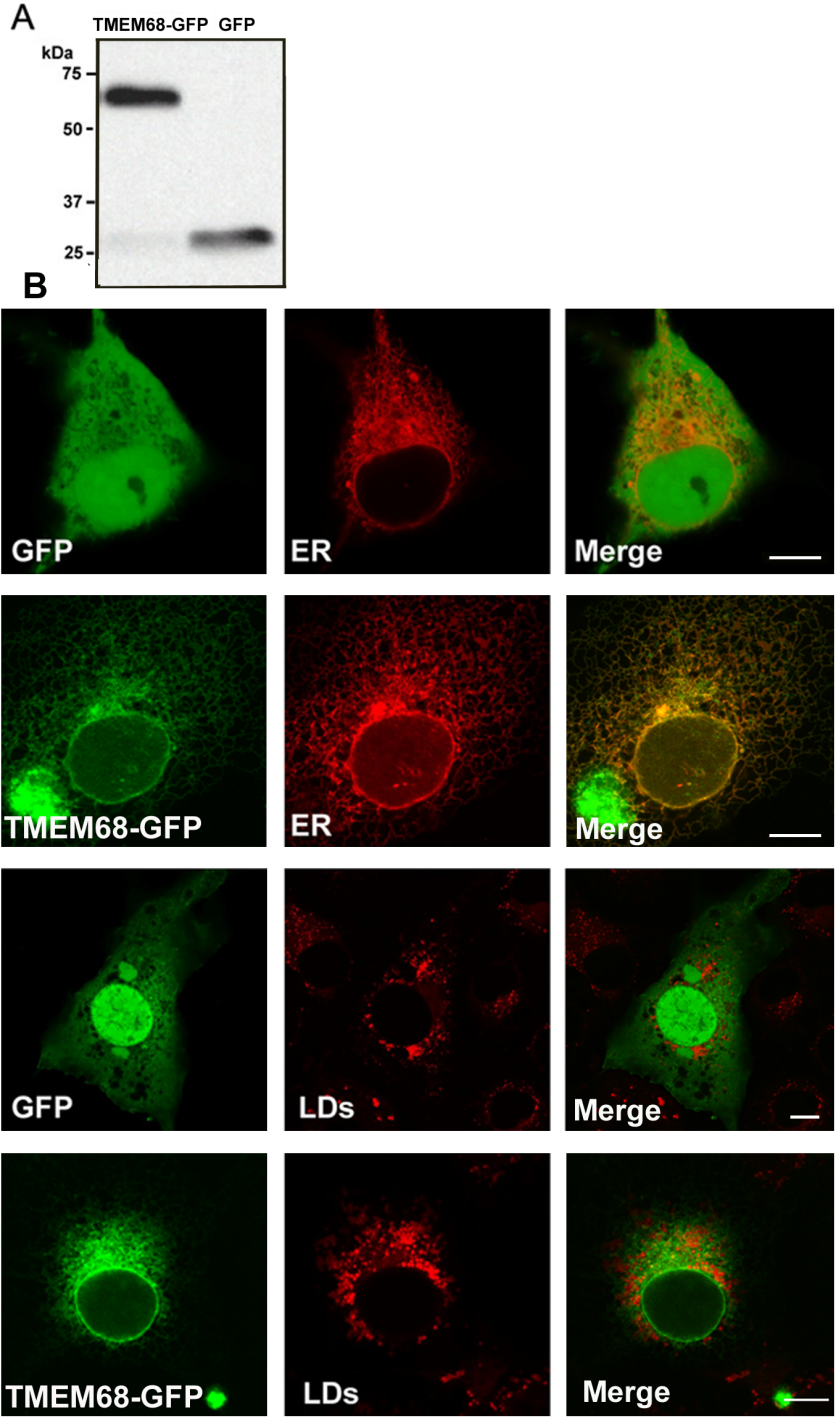
Subcellular localization of TMEM68-GFP. (A) Detection of TMEM68-GFP and GFP expression by immunoblotting. 48-h post transfection, cells were harvested, homogenized, and subjected to immunoblotting using an anti-GFP antibody. (B) Subcellular localization of TMEM68-GFP in mammalian cells. COS-7 cells were transfected with plasmids encoding for GFP, TMEM68-GFP, and DsRed-ER as indicated and imaged by confocal fluorescence microscopy. For the detection of LDs, cells were incubated with OA and stained with LipidTOX Deep Red. Scale bar = 10 μm. Figures are representative of three separate experiments.

Other members of the glycerophospholipid acyltransferase family have been shown to localize at different subcellular organelles such as mitochondria, endoplasmic reticulum, and lipid droplets. WoLF PSORT program analysis showed that TMEM68 may be mainly present in the ER (data not shown). To address the subcellular localization of TMEM68 more directly, we expressed a TMEM68-GFP fusion protein in COS-7 cells and performed confocal fluorescence microscopy. The expression of TMEM68-GFP and GFP was first confirmed by immunoblotting analysis (Fig 4A). Cells transfected with GFP or TMEM68-GFP expressed immunoreactive proteins of approximately 27 and 65 kDa, respectively, which is in accordance with the deduced MWs of both proteins. Next, we co-transfected COS-7 cells with TMEM68-GFP or GFP, and the ER marker DsRed-ER. As shown in Fig 4B, GFP exhibited a cytosolic and nuclear distribution but did not co-localize with the ER. In contrast, TMEM68-GFP displayed a reticular staining pattern and extensively co-localized with DsRed-ER suggesting that TMEM68 is indeed targeted to the ER in mammalian cells. Notably, when LD formation was increased by OA loading, TMEM68-GFP still exhibited a reticular distribution and did not become concentrated around LDs (Fig 4B). These results demonstrate that TMEM68 is localized at the ER, but not at LDs.

### The first TMD is required for targeting of TMEM68 to the ER

**Fig 5:**
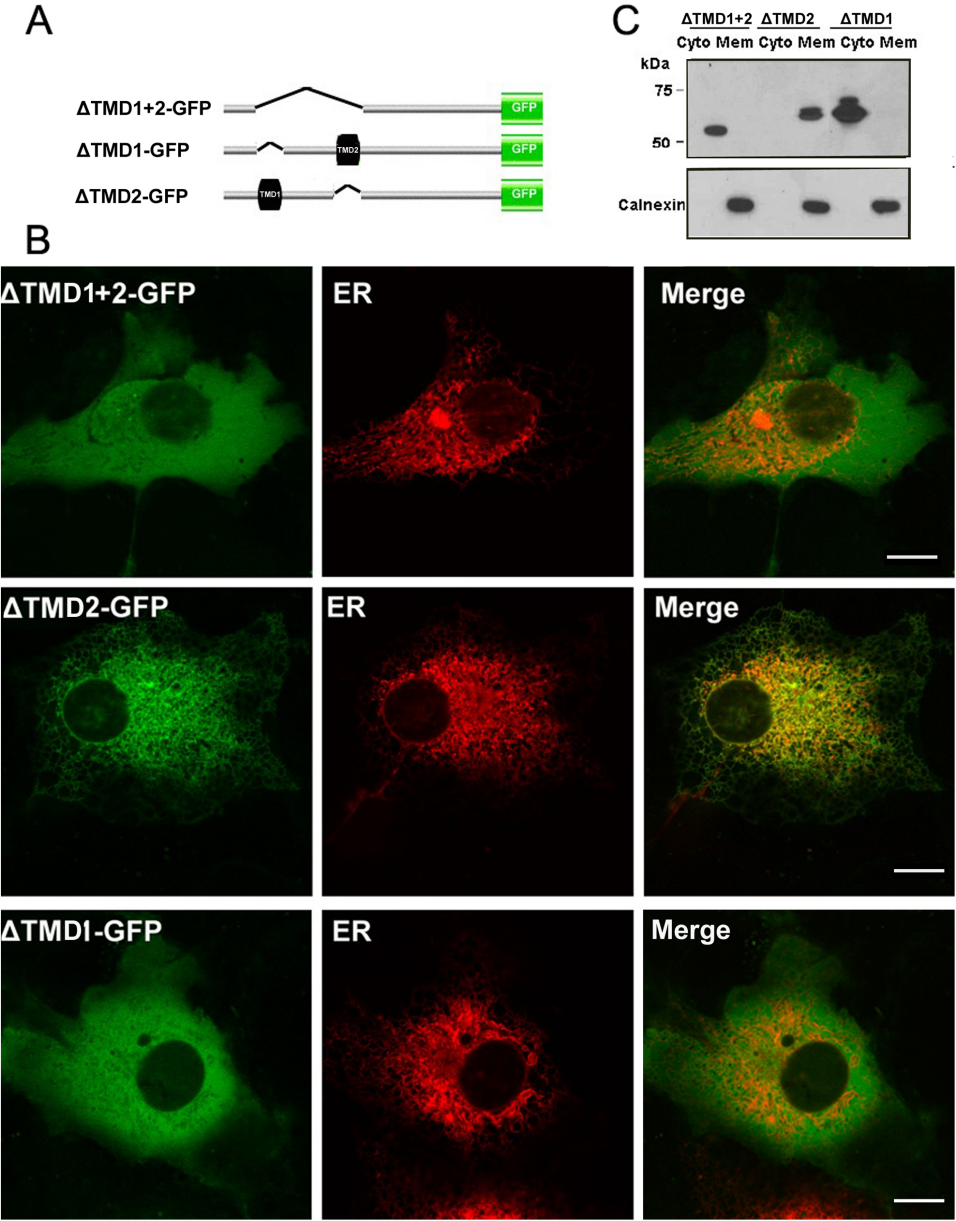
Contribution of TMDs to ER targeting of TMEM68. (A) Scheme of GFP-tagged TMEM68 mutant proteins harboring deletions of the first (ΔTMD1), the second (ΔTMD2) or both (ΔTMD1+2) TMDs (shown as black boxes) (B) COS-7 cells were co-transfected with constructs encoding for ΔTMD1-GFP, ΔTMD2-GFP or ΔTMD1+2-GFP and the ER marker, DsRed-ER. 48-h post transfection, cells were analyzed by a confocal laser scanning microscopy. Scale bar = 10 μm. Figures are representative of three separate experiments. (C) Post-nuclear supernatants of COS-7 cells expressing TMEM68 mutant proteins were fractionated by centrifugation into membrane (mem, pellet) and cytosolic (cyto, supernatant) fractions and analyzed by immunoblotting using antibodies against GFP or the ER marker calnexin.

We next assessed the functional relevance of the 2 putative TMDs for membrane association and subcellular positioning of TMEM68. To address which TMD is responsible for targeting of TMEM68 to the ER, we generated mutants lacking the first (ΔTMD1), the second (ΔTMD2) or both TMDs (ΔTMD1+2) (Fig 5A). The mutants and the ER maker, DsRed-ER, were co-expressed in the COS-7 cells and their subcellular localization was recorded by confocal fluorescence microscopy. As shown in Fig 5B, ΔTMD1+2-GFP displayed a diffused cytosolic distribution and did not co-localize with the ER indicating that the ER targeting signal resides within one or both TMDs. Notably, the mutant ΔTMD2-GFP, exhibited a typical ER staining pattern and co-localized with the ER marker (Fig 5B) whereas ΔTMD1-GFP was distributed as diffusely throughout the cytoplasm as ΔTMD1+2-GFP suggesting that TMD1 but not TMD2 confers association of TMEM68 to the ER. In order to confirm the imaging results subcellular fractionation experiments were performed. As shown in Fig 5C, ΔTMD1+2-GFP and ΔTMD1-GFP were recovered predominantly in the soluble fraction suggesting impaired membrane association of both mutants. In contrast, ΔTMD2 was recovered in the membrane fraction resembling the distribution of the integral ER-membrane protein calnexin. Taken together our results show that ER targeting of TMEM68 is dependent on the first but not the second TMD.

### The first TMD of TMEM68 is sufficient to target GFP to the ER

**Fig 6:**
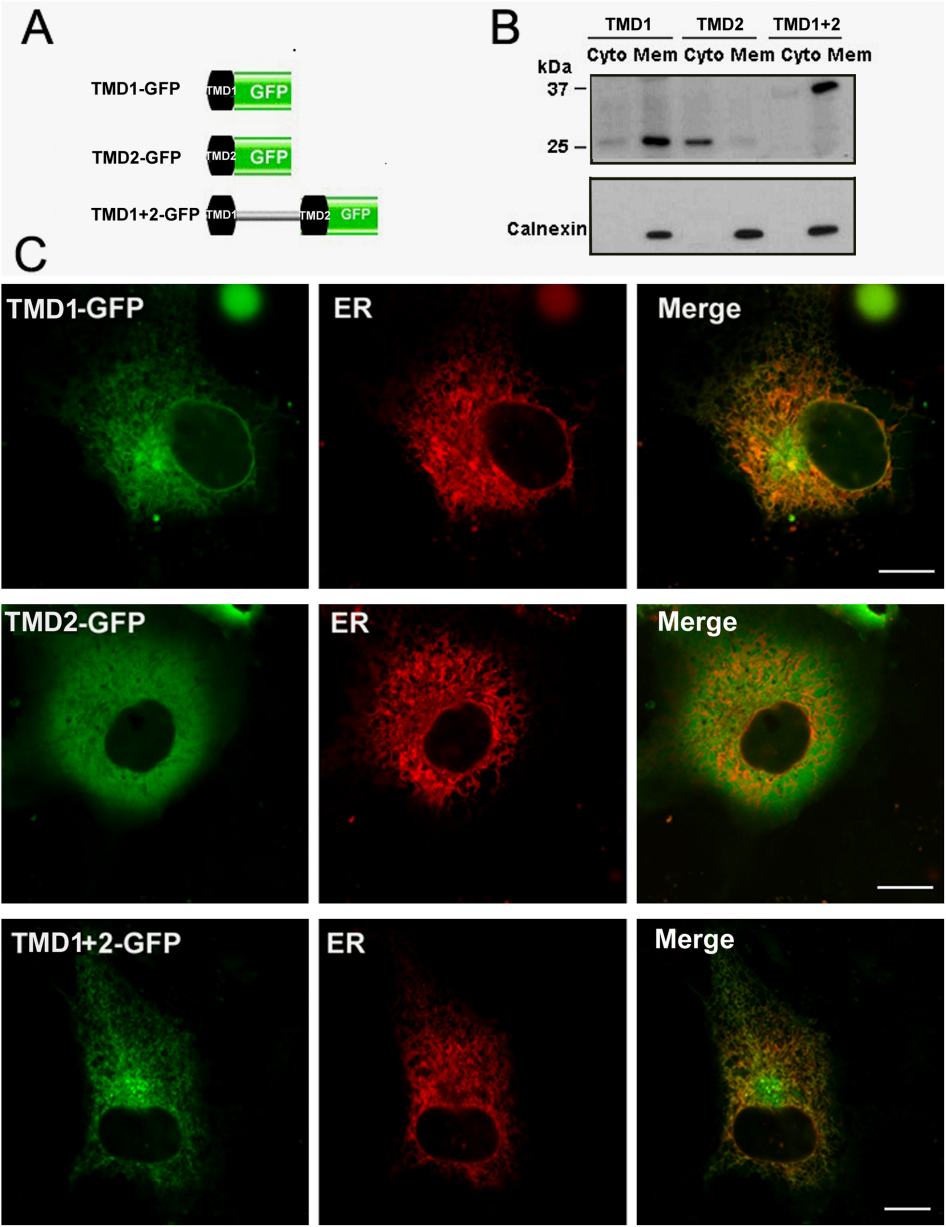
Targeting of GFP to the ER by the first TMD of TMEM68. (A) Scheme of the constructs expressing the first (TMD1), the second (TMD2), and both TMDs (TMD1+2) tagged with GFP. Black boxes represent the first and second TMDs of TMEM68. (B) Post-nuclear supernatants of COS-7 cells expressing TMD1-GFP, TMD2-GFP or TMD1+2-GFP were fractionated into cytosolic and membrane fractions and analyzed by immunoblotting using antibodies against GFP or the ER protein calnexin. (C) COS-7 cells were co-transfected with TMD1-GFP, TMD2-GFP or TMD1+2-GFP and the ER marker DsRed–ER as indicated and visualized by a confocal fluorescence microscopy. Scale bar = 10 μm. Figures are representative of three separate experiments.

Because a TMEM68 mutant lacking the first TMD is no longer localized at the ER an ER targeting signal may be present in this region. Thus, we asked whether the first TMD is sufficient to target cytosolic GFP to the ER. To this end, TMD1, TMD2 or TMD1+2 were expressed as GFP-fusion proteins (Fig 6A) in COS-7 cells and their subcellular distribution was assessed by subcellular fractionation and confocal fluorescence microscopy. As shown in Fig 6B, TMD1-GFP and TMD1+2-GFP were recovered predominantly in the membrane fraction resembling the ER marker calnexin whereas TMD2-GFP was found mainly in the cytosolic fraction.

Confocal fluorescence microscopy showed that TMD1+2-GFP and TMD1-GFP display a reticular staining pattern and co-localize with the ER marker, DsRed-ER (Fig 6C). In contrast, TMD2-GFP was distributed in a diffuse cytosolic staining pattern and did not co-localize with the ER. Thus, TMD1 but not TMD2 acts as an ER localization signal that is sufficient for ER targeting of cytosolic GFP.

### TMEM68 is highly expressed in adult brain

**Fig 7:**
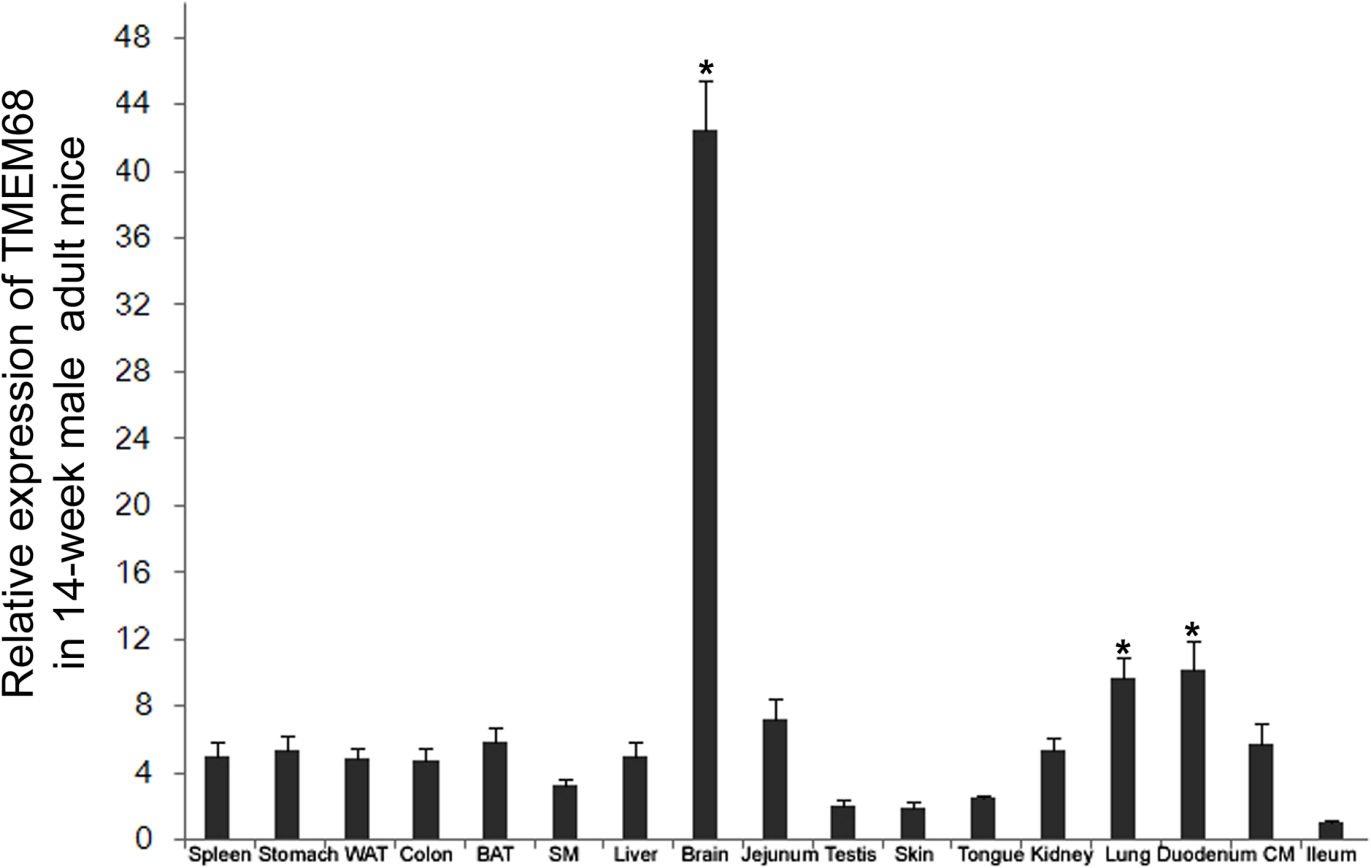
Expression of TMEM68 in adult murine tissues. Relative transcript levels of TMEM68 were measured by quantitative RT-qPCR using *36B4* as a reference gene. The y-axis represents relative mRNA expression levels of TMEM68. White adipose tissue (WAT), Brown adipose tissue (BAT), skeletal muscle (SM), cardiac muscle (CM). *, p<0.05; n = 3.

Finally, the mRNA expression of TMEM68 in 17 tissues of adult male C57BL/6J mice was examined by real time quantitative-PCR. As shown in Fig 7, TMEM68 mRNA was widely expressed in the tissues examined, although relative low levels were detected in tongue, testis, skin and ileum. However, the mRNA levels of TMEM68 were evidently higher in brain than in all other tissues tested. Thus, TMEM68 is highly expressed in the adult murine brain.

## Discussion

TMEM68 has been previously identified as a putative member of the glycerophospholipid acyltransferase family but its subcellular localization and enzymatic function(s) have not been addressed [10]. Our phylogenetic analysis of glycerophospholipid acyltransferases from mouse, human, and fruit fly shows that TMEM68 is evolutionarily highly conserved. Moreover, murine TMEM68 and its human and fly orthologues constitute a distinct subgroup within the glyerophospholipid acyltransferase family that is remotely related to other family members. The glycerophospholipid acyltransferase domain has been shown to contain 4 conserved motifs, which have been implicated in catalysis and substrate binding. Motif I is highly conserved among acyltransferase superfamily members and possesses a HXXXXD signature that is involved in catalysis [17]. Motif IV is the least conserved motif among the 4 acyltransferase motifs, of which the conserved proline is present in TMEM68. Phenylalanine and arginine residues in Motif II as well as glutamate and glycine residues in Motif III have been shown to be important for substrate binding in GPATs and AGPATs [11]. However, these residues are not well conserved in Motif II and III of murine TMEM68 suggesting that TMEM68 may not harbor GPAT or AGPAT activities. Phylogenetic analyses indicate a relationship of the TMEM68 subgroup to the DAG acyltransferase family (characterized by the pfam03982 domain) although sequence similarities with other diacylglycerol acyltransferase family members such as MGAT1 and MGAT2 are low. This suggests that TMEM68 may be a MGAT- or DGAT-like protein within the glycerophospholipid acyltransferase family. However, the absence of *in vitro* assay data precludes definite conclusions about the substrate specificity of TMEM68.

Consistent with the presence of two putative TMDs in TMEM68 biochemical analyses revealed that TMEM68 is strictly membrane associated when expressed in mammalian cells. Treatment with sodium carbonate, a reagent known to dissociate peripheral membrane proteins failed to release TMEM68 into the supernatant. In contrast, TMEM68 was extractable with detergents, such as SDS and Triton X-100 indicating that TMEM68 is an integral membrane protein. Protease protection assays demonstrated that both, the N- and C-terminus of TMEM68 are exposed to the cytosol. Together with the presence of 2 predicted TMDs this finding suggests that TMEM68 likely acquires a polytopic topology that leads to the orientation of both ends of the polypeptide towards the cytosol.

Consistent with the characteristic of a TM protein a TMEM68-GFP fusion protein displayed a reticular staining pattern and co-localized extensively with a recombinant ER marker. However, known ER targeting signals, such as an N-terminal signal sequence, an N-terminal di-arginine motif, or C-terminal di-lysine or KDEL retrieval motifs [18–20], are not present in TMEM68 suggesting that other motifs, e.g. the TMD, may determine ER localization of TMEM68. Indeed, confocal microscopy and biochemical fraction analysis indicated that a TMEM68 mutant lacking its two TMDs became a cytosolic protein and failed to associate with the ER membrane. Further results showed that already the loss of the first TMD (TMD1) compromised ER targeting and led to a cytosolic distribution of TMEM68. Moreover, TMD1 was sufficient to target cytosolic GFP to the ER whereas a mutant lacking TMD2 but not TMD1 was still able to associate with membranes and the ER. This suggests that TMD1 constitutes an ER targeting signal, which is both, required and sufficient for ER targeting of TMEM68. Until now, the process of directing membrane proteins to the ER by the TMDs has not been well understood. Both, the composition and length of TMDs seem to be important for the targeting to the ER and subsequent retention [21, 22]. Further studies are needed to determine the precise elements within TMD1 required for the localization of TMEM68 to the ER.

Several members of the glycerophospholipid acyltransferase superfamily, such as GPAT4, LPCAT1, LPCAT2, exhibit a dual localization at the ER and LDs [23, 24]. LDs are dynamic storage organelles for hydrophobic esters such as triacylglycerol and steryl esters and play pivotal roles in cellular energy homeostasis and lipid trafficking. In contrast to other organelles, which sequester luminal compartments by a lipid bilayer LDs have a hydrophobic core that is surrounded by a phospholipid monolayer [25]. This arrangement imposes specific requirements on the topology of proteins targeting the LD surface. The hydrophobic environment of the LD core precludes the presence of hydrophilic luminal domains in this compartment and is therefore not compatible with the presence of bi- or polytopic TMD proteins. In line with this notion, acyltransferases targeting LDs have been shown to acquire a monotopic topology via hairpin motifs or amphipathic helices that enables association with both, the ER and LDs [25]. In contrast to GPAT4 and other acyltransferases, TMEM68-GFP displayed a typical ER staining pattern and did not appear to associate with LDs even when LD formation was promoted by OA loading. This suggests that TMEM68 lacks a LD targeting motif or that its topology precludes association with the LD surface.

Members of glycerophospholipid acyltransferases family are highly expressed in tissues involved in energy metabolism or high lipid turnover, such as white adipose tissue, brown adipose tissue, skeletal muscle, cardiac muscle, liver, and testis [17]. However, specific members such as AGPAT4 and LPCAT4 have been shown to be mainly expressed in the brain [26, 27]. AGPAT4 has been characterized as LPAAT and was recently shown to regulate brain glycerolipid homeostasis [26]. Conversely, LPCAT4 was shown to lack LPAAT activity but to possess significant LPLAT activity towards other lysophospholipids such as LPC, LPE, and LPS [27]. The expression of selected glycerophospholipid acyltransferase members specifically in brain suggests that neuronal tissues harbor a distinct set of acyltransferase enzymes to regulate their membrane lipid composition. Notably, the expression pattern of TMEM68 closely resembles that of AGPAT4 and LPCAT4 with highest transcript levels in brain. Thus, TMEM68 may be part of a brain-specific set of acyltransferases implicated in neuronal glyerolipid homeostasis. The identification of potential substrates of TMEM68 *in vitro* and lipid analysis of TMEM68 mutant cells or animals is clearly warranted to shed light on the potential function of TMEM68 in glycero(phospho)lipid homeostasis.
